# The genomic architecture of continuous plumage colour variation in the European barn owl (*Tyto alba*)

**DOI:** 10.1098/rspb.2023.1995

**Published:** 2024-01-10

**Authors:** Tristan Cumer, Ana Paula Machado, Luis M. San-Jose, Anne-Lyse Ducrest, Céline Simon, Alexandre Roulin, Jérôme Goudet

**Affiliations:** ^1^ Department of Ecology and Evolution, University of Lausanne, Biophore Building, Lausanne CH-1015, Switzerland; ^2^ Laboratoire Évolution and Diversité Biologique, UMR 5174, CNRS, Université Toulouse III Paul Sabatier, Toulouse, France; ^3^ Swiss Institute of Bioinformatics, Lausanne, Switzerland

**Keywords:** continuous colour variation, selection, melanin, whole-genome sequencing, MC1R

## Abstract

The maintenance of colour variation in wild populations has long fascinated evolutionary biologists, although most studies have focused on discrete traits exhibiting rather simple inheritance patterns and genetic architectures. However, the study of continuous colour traits and their potentially oligo- or polygenic genetic bases remains rare in wild populations. We studied the genetics of the continuously varying white-to-rufous plumage coloration of the European barn owl (*Tyto alba*) using a genome-wide association approach on the whole-genome data of 75 individuals. We confirmed a mutation at the melanocortin-1-receptor gene (*MC1R)* is involved in the coloration and identified two new regions, located in super-scaffolds 9 and 42. The combination of the three regions explains most of the colour variation (80.37%, 95% credible interval 58.45–100%). One discovered region, located in the sex chromosome, differs between the most extreme colorations in owls sharing a specific *MC1R* genotype. This region may play a role in the colour sex dimorphism of this species, possibly in interaction with the autosomal *MC1R*. We thus provide insights into the genetic architecture of continuous colour variation, pointing to an oligogenic basis with potential epistatic effects among loci that should aid future studies understanding how continuous colour variation is maintained in nature.

## 1. Introduction

The variation of colour traits within a single species and often between populations has fascinated evolutionary biologists for decades because it questions how such (often heritable) variation is generated and maintained. For practical reasons, research on the genetic basis of animal coloration began and flourished on humans [1–3] and model systems (i.e. mice [4,5], but also domestic animals [6,7]), and has then been extended with the advent of genome-wide technologies to non-model species of birds [8–12], mammals [13,14], butterflies [15] and amphibians [16]. This has led to a better understanding of the genetics of coloration in the wild [17,18] although current research is still biasedly focused on colour traits exhibiting discrete variation and inheritance patterns (i.e. colour polymorphism) [11,13,14] or to the particular case of hybridizing species [8–10].

Colour traits often vary continuously between two extreme values within a single species and even populations of the same species, but this type of trait has been rarely studied [17,18]. Continuous colour variation often has a genetic origin [19] with a suspected polygenic or oligogenic basis [18]. The study of the genetics of continuous colour traits is challenging because they can be based on numerous variants of large to very small effect sizes but also because part of the continuous variation is non genetic but likely of environmental origin [20]. Further work is therefore needed to better characterize the molecular basis of the colour diversity observed in many species. Understanding the genetic basis of continuous colour traits should aid to better understand the role that their genetic architecture plays in maintaining such variation in natural contexts as well as in driving the covariation between coloration and other phenotypic aspects (e.g. behaviour, physiology) that explain the function of continuous colour variation in natural and sexual selection contexts.

**Figure 1.**
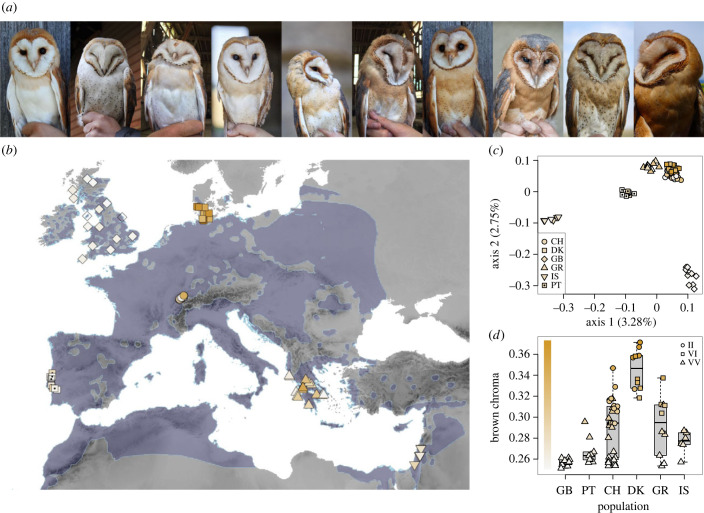
Variation in plumage coloration of the barn owl across Europe. (*a*) Pictures illustrating the plumage colour variation in Swiss barn owls. (*b*) Map of the samples used in this study. Each dot on the map corresponds to one individual, coloured accordingly to its own plumage colour. The type of dot matches the sampling localities in *c*. The current distribution of barn owls is plotted atop the map in blue (data from the International Union for Conservation of Nature (IUCN): BirdLife International 2019). (*c*) Genomic principal component analysis (PCA) including all sequenced individuals, with dots coloured according to individual plumage colour and shaped according to their sampling locality. (*b*) Boxplot summarizing variation in plumage coloration of the individuals from the different sampled localities. Brown chroma on the *y*-axis refers to the ratio of the red part of the spectrum (600–700 nm) over the complete visible spectrum (300–700 nm) of the reflectance of the feathers, a proxy of the redness of the individuals (see Material and methods section for details). Dots correspond to individuals, with shape matching the MC1R genotype and coloured according to plumage coloration. Picture credit, from left to right: ana Paula Machado (A.P.M.), Clément Grandjean—Terre&Nature, Guillaume Rapin (G.R.), Jeremy Bierer (J.B.), Daniel Aubort, G.R., A.P.M., J.B., Lukas Linder—Swiss Ornithological Institute, Isabelle Henry.

Barn owls (Tytonidae) are an ideal system to study continuous variation in coloration. In at least seven *Tyto* clades, the plumage of these cosmopolitan birds varies from white to dark rufous (figure 1*a*) owing to the differential deposition of melanin pigments [21–24]. In Europe, the species *Tyto alba* shows a pronounced south-to-north clinal variation (figure 1*b*), with individuals ranging from white in the south to dark rufous in the north of its continental distribution [25] (the British Isles are an exception to this trend, where owls are whiter despite inhabiting at high latitudes, a pattern attributed to drift; see [26] for details). A large variation in plumage coloration can also be found among individuals of the same locality (e.g. Switzerland, figure 1*d*). On the European continent, previous studies have shown that variation in the melanin-based coloration of the barn owl is associated with reproductive success and feeding rate [27], habitat choice [28], diet [29] wing morphology and stomach content while flying [30,31], as well as dispersal ability [32,33]. The association between coloration and a wide variety of traits might be induced by the pleiotropic control of the melanocortin system on numerous traits in addition to its central role [34] in the synthesis of melanin pigments [21,22]. Plumage coloration in the barn owl is strongly heritable (*h*^2^ of the ventral plumage coloration of owls ranges from 0.57 to 0.84; [35,36]) and dependent on the melanocortin system. A diallelic mutation (*V126I*) in the melanocortin-1 receptor gene (*MC1R*) explains a large proportion of the phenotypic variance in plumage coloration [37], with individuals homozygous for the *MC1R* allele with a valine at position 126 (allele denoted *MC1R-white*, genotype *MC1R_VV_* below) being white to light rufous, and individuals with at least one isoleucine being dark rufous (allele denoted *MC1R-rufous*, genotypes *MC1R_VI_* and *MC1R_II_* below).

The mechanisms underlying the maintenance of plumage colour variation in the barn owl at a local and continental scale remain elusive. To date, three non-mutually exclusive mechanisms have been proposed. At a continental scale, plumage coloration may be a primary target of local adaptation, as suggested by the fact that phenotypic differentiation across the European cline is much more pronounced than neutral genetic differentiation [25,38–40]. The local adaptation hypothesis is further supported by the higher frequency of the *MC1R-rufous* allele in the north of the distribution, whereas it is nearly absent in the south [25]. Local adaptation might be triggered by the presence of different prey species along the cline [29] and differences in foraging strategies associated with plumage coloration [41]. Other factors might also be at play: for instance, plumage coloration might be adapted to local humidity levels in order to minimize plumage bacterial degradation or to maximize camouflage [23]. At a local scale, density-dependent selection on the different morphs may maintain colour variation as pointed out by Kvalnes *et al*. [37] who showed that low breeding densities selected for more rufous females, whereas high densities selected for whiter females. In addition, sexually antagonistic selection may also be acting at a local scale and be responsible of maintaining colour variation. Dark melanic females (i.e. harbouring a more rufous plumage with many black spots located at the tip of the ventral body feathers) were sexually mature earlier and survived better than lighter melanic females. However, lighter melanic males (i.e. harbouring a whiter plumage with few black spots located at the tip of the ventral body feathers) were sexually mature earlier and survived better than darker melanic males [42,43]. Genetic mechanisms can also be at play and aid maintaining colour variation. Using quantitative genetic analysis, we detected a potential epistatic effect of the *MC1R-rufous* allele. This variant seems to mask (and thereby hide from selection) the expression of other colour variants, which are expressed in owls carrying the *MC1R-white* allele [36]. Thus, conclusive evidence for the selective targets and agents driving colour variation at a local or continental scale are still amiss in the European barn owl. Identifying the gene(s) underlying continuous plumage colour variation and their effects is a first step to unravel the molecular basis of this variation.

In the present study, we investigated the genomic basis of continuous colour variation in the European barn owl (*T. alba*). By exploiting whole-genome data of 75 barn owls from six different localities in Europe and the Middle East, combined with spectrophotometric data on their coloration we: (i) identified major Quantitative Trait Loci (QTL), (ii) studied the levels of variation explained by these QTL variants, (iii) discuss the potential functional role of these loci in the melanic pathway and in building up associations between coloration and other phenotypic traits, and (iv) discuss how our findings could be used to better understand the mechanisms responsible for the maintenance of continuous colour variation in the European barn owl.

## 2. Results and discussion

### (a) Genomic and phenotypic landscape of the European barn owl (*Tyto alba*)

We focused our study on European barn owls from the western Palaearctic. In our sampling, plumage coloration varies from white in the south (in the Iberian Peninsula: Portugal, PT, and Levant: Israel, IS) to dark rufous in the north (Denmark, DK) (figure 1*b–d*). Two localities (Switzerland, CH, and Greece, GR) harboured a high colour diversity (figure 1*a–d*), and we also included owls from Great Britain (GB), which are characterized by their whiter plumage coloration (figure 1*b–d*). We conducted whole-genome sequencing of 75 individuals from these localities, yielding a total of 5 112 936 single-nucleotide polymorphisms (SNPs) after filtering. Neutral PCA supported genetic differentiation among localities (figure 1*c*, see also [40]), with the first axis separating the Levant lineage (IS) from the rest, and the second axis mainly separating individuals from GB from the rest of the European localities (electronic supplementary material, figure S2).

In line with the results of San-Jose *et al.* [36], *MC1R* genotype extracted from the whole-genome sequencing data of the individuals was consistent with their plumage coloration, with the *MC1R-white* allele (V) in populations with a white phenotype, and the *MC1R-rufous* allele (I) present in all populations with rufous individuals, and an increased frequency in the northern population, with only *MC1R_VI_* or *MC1R_VV_* individuals in DK (figure 1*c*). These results are also in line with the known distribution of this allele at the European scale [25].

### (b) Genome-wide association identifies a new autosomal region associated with plumage coloration

**Figure 2.**
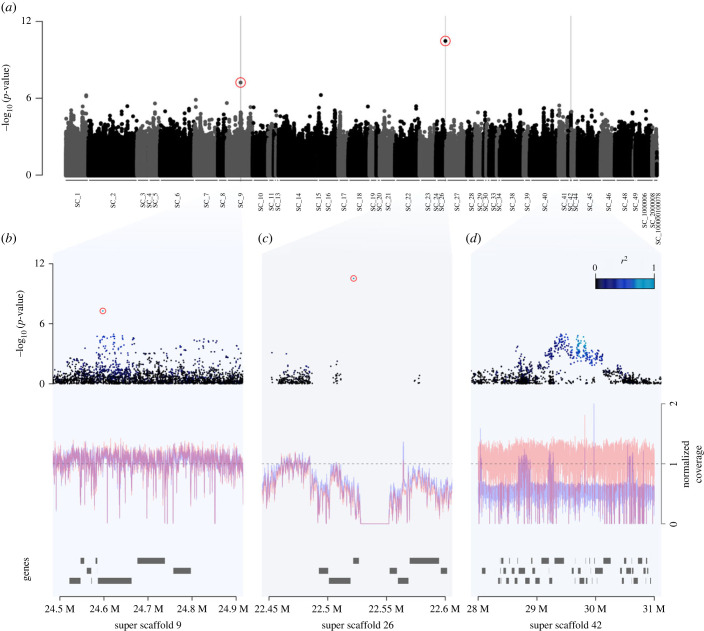
Genome-wide association (GWA) study between the genotypes of the barn owl and plumage coloration. (*a*) Association scores (-log_10_[*p*-values]) of each SNP along the genome with plumage coloration in all European samples. Alternated colours depict the successive super-scaffolds (SC_) in the genome. Significant SNPs (presenting a deviation from the 1 : 1 line of the qq-plot; electronic supplementary material, figure S4) are highlighted by surrounding red circles. Grey bars highlight the regions of interest presented in (*b*), (*c*) and (*d*). (*b,c,d*) Zooms on the different regions of the genome displaying a strong signal of association with plumage coloration. Upper panels show the association scores of each SNP within the regions of interest. The colour of the dots represents their level of association with the focal SNP within each region (colour scale is given in *d*). Red circles surround the two outlier SNPs detected in the GWAS. The red and blue lines represent the mean normalized coverage for males and females, respectively, while the dashed line represents the expected normalized coverage. Rectangles below represent the genes annotated in the different regions.

We used a genome-wide association (GWA) approach to identify SNPs associated with plumage coloration across all samples and localities (figure 2). The inclusion of the genetic relatedness matrix in the model successfully controlled for population stratification, as supported by the low genomic inflation factor (*λ* = 1.05) and the alignment of most SNPs along the 1 : 1 *qq*-line (electronic supplementary material, figure S4) [44].

The GWA identified one outlying SNP at the genome-wide significance level (figure 2*a*). This outlying variant was the variant identified by San-Jose *et al.* [36], located at the *MC1R* gene (hereafter *MC1R variant*, G -> A, located in the position 22 522 039 of super-scaffold 26), with an association score of 5.703 × 10^−11^ (figure 2*a* and *c*, a score smaller than Bonferroni's significance threshold: 0.05 / 5 112 936 tests = 9.779117×10^−09^). This is the *MC1R* variant previously discovered using a candidate gene approach [22]. Our genomic data shows that the signal in this region was not expanded by linkage disequilibrium, LD, to the surrounding variants (all showing a relatively low association with coloration and little LD among them; figure 2*c*). Although this finding might indicate the absence of strong, recent selection [45], the low coverage in this region (probably due to its high GC content [22], see coverage fluctuation in panel 2*c*), is likely to explain the low number and sparse distribution of SNPs around the discovered variant. Thus, inferences about selection in this region should be cautiously done.

The second locus highlighted in figure 2*a* was located at the *MATN2* gene (hereafter, *MATN2 variant*, A -> G, position 24 597 481 of super-scaffold 9). It had an association score of 8.813 × 10^−08^ (figure 2*a* and *b*, non-significant at the Bonferroni threshold of 9.779117 × 10^−09^) and it could be a potential type 2 error. This SNPs nevertheless clearly deviated from the 1 : 1 line of the qq-plot (electronic supplementary material, figure S4). Moreover, a randomization test supported that our GWA was not prone to type 2 error signals (i.e. association scores as high or higher than the one of the *MATN2* variant had a low probability (28 out of 1000 iterations yielded SNPs with an association score equal or lower than the association score of this locus, i.e. *p* < 8.813×10^−08^)). Although the strongest signal in the region came from an intronic region of the *MATN2* gene, a cluster of SNPs in linkage disequilibrium tended to associate with plumage coloration (figure 2*c*) including multiple SNPs near the *MTDH* gene, located 160 kilobase pair (kbp) downstream of the *MATN2* variant.

### (c) Stratified analysis of the Swiss population pinpoints a region on the Z chromosome

Previous studies suggested a major role of the *MC1R* gene in plumage colour variation in the barn owl, with potential epistatic effects with other genetic variants [36]. Quantitative genetic studies suggest that the *MC1R-white* allele permits the expression of further genetic variation for plumage coloration, whereas the alternative allele, *MC1R-rufous*, seems to mask the effect of other genetic variants. Because such an expected epistatic effect can hinder QTL discovery (mainly based on additive effects), we narrowed down our analysis to consider only the Swiss individuals in our dataset. Barn owls from this locality were chosen because the potential *MC1R* epistatic effect was detected in Swiss owls, it is one of the populations with the highest variation in coloration (figure 1*b*) and there is no apparent genetic structure at the whole-genome scale associated with coloration nor the *MC1R* locus [40]. Indeed, a neutral PCA confirmed no differentiation according to coloration nor *MC1R* genotype among Swiss individuals (electronic supplementary material, figure S3). Whole-genome F_IS_ was 0.005 and whole-genome F_ST_ between *MC1R_VV_* and *MC1R_II_* individuals was 0.0002, which is consistent with an absence of substructure.

Because of the reduced dataset, we used F_ST_ scans rather than the mixed modelling approach conducted above for the complete set of samples. We first scanned for highly differentiated genomic regions between the whitest (brown chroma, BC < 0.28, *N* = 10) and the most rufous (BC > 0.28, *N* = 20) individuals without considering their *MC1R* genotype. This first scan showed no strong signal of differentiation along the genome (electronic supplementary material, figure S6). We then focused only on the 18 Swiss barn owl homozygotes for the *MC1R-white* allele (i.e. *MC1R_VV_* individuals), as we expected this variant to allow the expression (and detection) of the effects of other genetic variants on plumage coloration [36]. We scanned for highly differentiated genomic regions between the whitest (BC < 0.28, *N* = 10) and the most rufous (BC > 0.28, *N* = 8) *MC1R_VV_* owls and this revealed one highly differentiated region (F_ST_ > 0.8) located on the sex chromosome (electronic supplementary material, figure S7a and S7b). In the GWA across all localities, the *Z variant* was not significantly associated with coloration although a cluster of SNPs in LD within this region shows a clear tendency to be associated with plumage coloration (figure 1*d*). The association between sex-linked variation and plumage coloration is expected given that previous quantitative genetic studies found that a substantial part of genetic colour variation is harboured by the Z chromosome [35]. The highlighted region includes multiple genes (electronic supplementary material, table S3), and among them: *CHRBP* (LOC104362934)*.* We selected the most differentiated variant within this region to infer the genotype at this locus for downstream analyses (hereafter called *Z variant*, G -> A, position 29 829 678 of super-scaffold 42).

### (d) Contribution of the discovered loci to plumage colour variation

**Figure 3.**
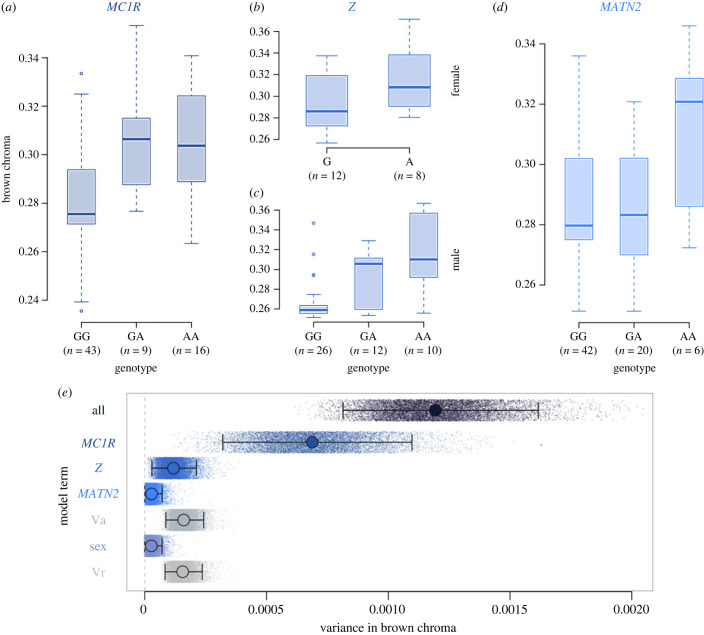
Estimation of the contribution of the different loci to plumage coloration. (*a*–*d*) Boxplots of the relationship between the genotype at the different loci and plumage coloration. (*a*) *MC1R* variant, (*b*) *Z* variant for females, (*c*) *Z* variant for males and (*d*) *MATN2* variant. (*e*) Results of the animal model partitioning plumage colour variation into the three loci, sex, the remaining additive genetic variance (Va) and residual variance (Vr). Small dots represent variance values of each term at each model iteration. Large circles indicate the mean posterior variance and 95% credible intervals (black lines).

In order to estimate the contribution of each of the three loci identified above (the *MC1R, MATN2* and the *Z* variants) to plumage coloration, we fitted an animal model allowing to also estimate the fraction of additive genetic variance that remains unexplained (figure 3*b*). Overall heritability of plumage coloration was 0.8 (95% credible interval, CrI: 0.58–1.05). The *MC1R* locus had the largest effect on coloration (proportion of variance explained: 0.69, 95% credible interval, CrI: 0.42–0.93), in line with previous studies focused on a large sampling of Swiss individuals [22,37]. The *Z* locus had a smaller, yet non-negligible effect on coloration (0.09, 95% CrI: 0.03–0.17), while the *MATN2* locus had the smallest effect with the lower 95% CrI close to zero (0.02, 95% CrI: less than 0.01–0.06). Moreover, models' deviance information criterion (DIC) values did not support that including the effect of the *MATN2* locus had a substantial impact on explaining colour variation (ΔDIC = 0.69) contrary to the other loci (ΔDIC*_MC1R_* 54.66, *Δ*DIC*_Z_*_locus_ = 22.03). We thus remain cautious about the role that the *MATN2* locus has in the plumage coloration of barn owls, despite the signal observed in the GWA. Further investigation including more individuals and functional validation should allow us to verify the association of the *MATN2* locus and barn owl coloration. We also detected that a non-negligible amount of colour variation (0.12, 95% CrI: 0.07–0.19) can still be attributed to genetic variants yet to be discovered. These variants are likely to have a smaller effect on coloration than the variants highlighted in this study. Despite the remaining work to better identify the genetic architecture sustaining coloration in the barn owl, our study finds support for an oligogenic architecture (based on few variants of major effect, particularly the *MC1R* locus) underlying continuous plumage coloration in the barn owl.

### (e) Dominance, additive effect, linkage and epistasis

The relationship between the genotypes at these three loci and plumage coloration (figure 3*a–d*) informs us about the dominance interactions between the alleles within these loci. For both *MC1R* and *MATN2* heterozygotes seem to have a similar trait value as one of the homozygotes, suggesting dominance of one of the alleles over the other. However, the direction of the dominance is in opposite directions for both loci, with a dominance of the rufous allele at the *MC1R* locus and a dominance of the white allele at the *MATN2* locus. On the other hand, the *Z* locus seems to harbour an additive effect between the two alleles in males (diploids), with heterozygous individuals displaying an intermediate phenotype relative to the two homozygotes.

The association of different loci with a phenotypic trait can be due to physical linkage between them when placed on the same chromosome [46]. The three loci we identified are located on independent scaffolds of the assembly of the barn owl genome (super-scaffolds 9, 26 and 42 for the *MATN2*, *MC1R* and the *Z variant*, respectively). The location of these regions in other birds’ genomes (assembled at chromosome level), shows that they are also located in distinct chromosomes (electronic supplementary material, table S4). These results, consistent with the known conserved synteny of bird genomes [47], support the physical independence of these three loci. The strong correlation detected between *MC1R* and *MATN2* variants (*r* = 0.67) may thus be due to selection on plumage coloration [25]. The contribution of the three loci to plumage coloration (see *Contribution of multiple loci to plumage colour variation* section for details) summed to their different dominance effects within each locus, suggests that the combined genotypes at these loci might be sufficient to give a broad, continuous panel of plumage coloration even if the combined effects of the three loci were only additive. Epistatic effects such as those hypothesized in the literature [36] and that our analysis seems to support (see *Stratified design in the Swiss population pinpoints a region on the Z chromosome*) could even amplify the range of possible phenotypes. However, the sampling size of this study limits our capacity to study such interactions in detail. Further research with an expanded dataset should allow us to measure how these three loci interact to build continuous plumage coloration in the barn owl.

### (f) Potential role of the newly discovered loci in the melanin synthesis pathway

The genomic regions identified in this study harboured genes putatively involved in melanin synthesis and thus in determining plumage coloration in the barn owl. Eumelanin and pheomelanin are the two pigments responsible for variation in plumage coloration in the barn owl [48]. The synthesis of one pigment or the other from the same precursor (tyrosine) relies on a series of reactions that are ultimately catalysed by specific melanogenic enzymes (TYR, TYRP1, TYRP2). These are all regulated by the MITF transcription factor, which is itself regulated by several signalling pathways known to influence coloration in vertebrates (including MAPK, WNT, PKC and cAMP pathways) [49]. *MTDH*, a gene located near the discovered *MATN2* variant, has been connected to both MAPK and WNT pathways, notably by downregulating ERK1/2 signalling [50]. Although the molecular implications of *MTDH* in coloration are still barely understood, variation affecting this gene may interfere with the regulation of MITF and thus impact plumage coloration.

Among the pathways regulating MITF, the cAMP pathway is activated by the binding of α-MSH (melanocyte-stimulating hormone) to MC1R, triggering the synthesis of melanin [49]. The effect of the *MC1R* gene in the coloration of the barn owl has been previously discussed in detail [22]. The peptidic hormone α-MSH is produced through the cleavage of the pro-opiomelanocortin protein, encoded by the *POMC* gene, whose transcription can be activated by the binding of CRH (corticotropin-stimulating hormone) to its receptor CRHR1 [51]. The discovered *Z* variant is in the vicinity of the *CRHBP* gene (the variant is located 63 501 bp downstream of the gene), which codes the inhibitor of CRH: the CRH binding protein (CRHBP), which may have the potential to influence *POMC* expression and thereby plumage coloration. A colour variant directly impacting *POMC* expression may also affect the expression of other phenotypic traits, given the known pleiotropic effects of the melanocortin system on coloration but also on behaviour, metabolism and on different hormonal systems [34]. CRH as well as POMC-derived hormones are main regulators of the stress response [51], which has been previously observed to differ among individuals displaying different colorations in barn owls as well as in other vertebrate species [52]. Thus, further research on the molecular basis of colour variation in the barn owl may offer new insights to understand how associations among distinct phenotypes evolves.

### (g) Maintenance of continuous colour variation in the European barn owl

Three non-mutually exclusive mechanisms have been proposed to explain the maintenance of continuous variation in plumage coloration in barn owls. At a large European continental scale, coloration might play a role in local adaptation [25,38,39], while at the local scale, coloration might be under density-dependent selection [37] and/or sexually antagonistic selection [42]. The clearer genomic architecture of plumage coloration described in the present study opens new avenues to formally test these hypotheses, given that the different proposed mechanisms of selection can leave traces at the genomic scale. The local adaptation hypothesis could be tested in the future by measuring traces of selection around the discovered variants. A larger number of samples, SNP density and improved assembly of the GC-rich region around the MC1R will be needed. Additionally, digging into the specific history of the genomic regions that are associated with coloration would also allow reconstructing their evolutionary history at the continental scale, compare it with the neutral history of the populations and test different selection scenarios [39,53].

Testing the two other hypotheses (namely, density-dependent and sexually antagonistic selection) will require the combination of both genomic and fitness data. Density-dependent selection should leave traces at the genomic level. If more rufous individuals are positively selected at low breeding densities [37], rufous variants will be expected to associate with a higher fitness and to have their frequency increasing over time until breeding density increases enough for white variants to be positively selected. The sexually antagonist selection hypothesis could also be tested by looking at how the association of the genomic regions associated with plumage coloration and fitness proxies changes between the sexes [54]. At the genomic level, sexually antagonistic selection generates intra-locus sexual conflict, which is thought to be resolved through the evolution of sexual chromosomes [55]. Here, we observed that part of plumage colour variation is located on the Z chromosome. This should lighten antagonistic selection but, as we showed here, sex-linked variation for coloration is proportionally smaller than autosomal variation and, moreover, sex-linked variation might epistatically interact with autosomal variation (with the *MC1R* locus). The genetic architecture of coloration might thus impose constraints to solve the sexual conflict, maintaining plumage colour variation within both sexes and making the evolution of sexual dimorphism difficult.

## 3. Conclusion

Using a genome-wide association study, we identified two new regions potentially influencing plumage coloration of the European barn owl and confirmed the major role of a previously discovered variant at the *MC1R* gene. This study constitutes a first step to understand the molecular basis of continuous variation in coloration and suggests an oligogenic architecture underlying this trait in the barn owl. Indeed, combining quantitative genetics with discoveries from GWA, we showed that a large part of continuous colour variation can be explained by a few markers. Further analyses aiming to pinpoint the causal mutations, their effects and interactions in building colour variation and to clarify their past and recent evolutionary history of these loci should allow a better understanding of the maintenance of continuous colour variation. Our study also opens new avenues to understand how colour variation in melanin-based traits relates to other aspects of the phenotype, namely stress response, a relationship observed across different vertebrate taxa and that might have its basis on the pleiotropic action of the melanocortin system. Because several (sub)species of the Tytonidae family exhibit plumage colour clines at a continental scale in America, Oceania and Africa, it would be interesting to investigate if the discovered loci are also involved in the plumage coloration of these other taxa and whether continuous variation has convergently evolved in this group. At a broader scale, this study exemplifies the possibility of a better understanding of the genetic basis of continuous traits, and it thus provides an opportunity to better characterize the relationship of the triptych genotype - phenotype - environment and thereby to build bridges between the ecology and the evolution of wild species.

## 4. Material and methods

### (a) Sampling design, sequencing and SNPs calling

#### (i) Sampling

In order to cover the phenotypic range of plumage colour variation of the barn owl in Europe, we used the whole genomes of 55 owls from six localities (electronic supplementary material, table S1, figure 1*b*): nine individuals from Portugal (PT), 10 from DK, 10 from GB, 10 from GR, 6 individuals from IS and 10 individuals from Switzerland (CH) (see Machado *et al*. [26] and Cumer *et al*. [40]). The sampling was extended with additional sequencing of 20 individuals from Switzerland (CH), where a large colour variation exists (figure 1*d*). These complementary individuals were selected to be mostly homozygous for the *MC1R* genotype (either VV or II) and preferably males (based on the sexing described by San-Jose *et al*. [36]) in order to reduce the effect of sexual differential in plumage coloration (described by [25]). *MC1R_VV_* individuals were also selected to cover the wide range of coloration variation possible within this genotype [36]. In the final dataset, the Swiss population was represented by 30 individuals including 18 MC1R_VV_, 11 MC1R_II_ and one MC1R_VI_ individuals. Among the MC1R_VV_, 10 were considered as white and 8 rufous (brown chroma of the reflectance spectra less than 0.28 and greater than 0.28, respectively, see *Phenotypic measurement* section of the Material and methods for details, electronic supplementary material, table S1).

#### (ii) DNA extraction and sequencing

For these new 20 individuals, we followed a similar library preparation and sequencing protocol as described in Machado *et al*. [26]. In brief, genomic DNA was extracted using the DNeasy Blood & Tissue kit (Qiagen, Hilden, Germany), and individually tagged. In total, 100 bp TruSeq DNA PCR-free libraries (Illumina) were prepared according to the manufacturer's instructions. Whole-genome resequencing was performed on multiplexed libraries with Illumina HiSeq 2500 PE high-throughput sequencing at the Lausanne Genomic Technologies Facility (GTF, University of Lausanne, Switzerland).

#### (iii) SNP calling

The bioinformatics pipeline used to obtain analysis-ready SNPs for the dataset including the 75 individuals was adapted from the Genome Analysis Toolkit (GATK) Best Practices [56] to a non-model organism following the developers' instructions, as in Machado *et al*. [26]. In brief, raw reads were trimmed with Trimommatic v.0.36 [57] and aligned to the reference barn owl genome (National Center for Biotechnology Information (NCBI) RefSeq assembly: GCF_018691265.1, scaffold level assembly, described in Machado *et al.* [26]) with BWA-MEM v.0.7.15 [58]. Base quality score recalibration (BQSR) was performed using high-confidence calls (also referred to as ‘true variants’) obtained in Cumer *et al*. [40] and following the standard procedure described in Machado *et al*. [26]. Genotype calls were then filtered for analyses using a hard-filtering approach using GATK and VCFtools v0.1.14 [59]. Calls were thus removed if they presented: low individual quality per depth (QD < 5), extreme coverage (800 > DP > 2000) or mapping quality (MQ < 40 and MQ > 70), extreme hetero or homozygosity (ExcessHet > 20 and InbreedingCoeff > 0.9) and high read strand bias (FS > 60 and SOR > 3). We then removed calls for which up to 5% of genotypes had low quality (GQ < 20) and extreme coverage (GenDP < 10 and GenDP > 40). We then filtered to retain only bi-allelic loci, yielding a dataset of 10′608′379 SNPs. For downstream analyses, SNPs were finally filtered for a minor allele frequency higher or equal to 0.05, yielding to a final dataset of 5 112 936 SNPs.

In downstream analyses, genes present in specific regions were identified using the annotation of the reference genome provided by the NCBI (NCBI Tyto alba Annotation Release 102, available at https://ftp.ncbi.nlm.nih.gov/genomes/all/annotation_releases/56313/102/).

### (b) Phenotypic information

#### (i) Sex determination based on WGS data

When unknown, individual sex was assessed using whole genome information. Mean SNP coverage for autosome (Super-Scaffold 1) and Z chromosome (Super-Scaffold 42) [26] were extracted using VCFtools v0.1.14 [59]. The ratio of mean coverage at the Z chromosome over the autosomal region allowed the identification of two distinct groups of individuals, with a ratio close to one for males and 0.5 for females (electronic supplementary material, figure S1, individual sex based on WGS is reported in electronic supplementary material, table S1) [60].

#### (ii) Plumage colour assessment measurements

Plumage coloration was measured by calculating the brown chroma index from reflectance spectra (see Antoniazza *et al.* [38] and Machado *et al.* [26] for details). Briefly, the brown chroma represents the ratio of the red part of the spectrum (600–700 nm) over the complete visible spectrum (300–700 nm) of the reflectance spectrum, with higher values indicating darker rufous colorations. The reflectance of four points of the top of three overlapping breast feathers was measured using a S2000 spectrophotometer (Ocean Optics) and a dual deuterium and halogen 2000 light source (Mikropackan, Mikropack). An individual's brown chroma score was obtained as the average of these points. This method correlates with observational assessments using colour chips (*r* = –0.78, *p* < 0.0001) [61] and it is highly repeatable (97.6%) [38].

### (c) Neutral diversity, population structure and phenotypic distribution

#### (i) GRM, kinship matrix and PCA

The individual-based relatedness (*β*) [62] and inbreeding coefficient was calculated with the package *SNPRelate* [63] in R (v4.2.2, [64]) for all 75 individuals. In order to avoid redundant signal from linked SNPs, rare (minor allele frequency (MAF) < 0.05) alleles were discarded and we trimmed the dataset to only retain SNPs with a linkage disequilibrium *r*^2^ lower than 0.4 (computed over a maximum of 500 kbp using the *LD.thin()* function from the *gaston* package [65]), yielding a total of 1′033′866 SNPs for analysis. The kinship matrix was then transformed into a Genetic Relationship Matrix (GRM) using the *kinship2grm()* function from the *hierfstat* package [66]. We assessed population structure using principal component analyses (PCA) (*SNPRelate* [63]), one on the entire dataset and only on the 30 Swiss individuals. Population pairwise *F*_ST_ [62] was also computed with the *hierfstat* package [66], and results are reported in electronic supplementary material, table S2.

### (d) Identification of genomic regions associated with plumage coloration

#### (i) GWAS on European samples

To test for associations between genotypes and plumage coloration of the 75 European individuals, we used the association.test() function in the *gaston* package [58] fitting a linear mixed model (LMM) using the average information restricted maximum likelihood (AI-REML) algorithm (as implemented in the lmm.aireml() function in the *gaston* package [65]). The model included the sex of the individuals as a covariable and the GRM as a random effect to account for population structure and cryptic relatedness. We used the Wald test to assess the strength of the association between SNPs and phenotypes. The significance threshold of the tests' *p*-values was adjusted following the Bonferroni correction [67]. Test significance was also visually assessed based on the SNPs deviation from the 1 : 1 line in the qq-plots. Results of the GWAS are presented in figure 2. A zoom on all the genes related to the melanin pathway present in the assembly (identified by Machado *et al.* [58]) is presented in electronic supplementary material, figure S5. To validate the association between plumage coloration and the SNPs, we repeated the GWA but randomizing the phenotype (plumage coloration) among all the individuals at each iteration (*N* = 1000 iterations). If the GWA signal is robust to false positive due to population stratification, we expect the association scores of SNPs from each iteration to be above the significance threshold of 0.05 adjusted according to Bonferroni's correction. Across our 1000 randomized analyses, we did not find any SNP above the Bonferroni threshold.

#### (ii) F_ST_ scans within Swiss individuals

In order to detect other loci involved in the coloration of the barn owl, we ran pairwise F_ST_ using the *snpgdsFst()* function in *SNPRelate* package [63]. Scans contrasted (i) rufous (i.e. Spectro > 0.28; *N* = 20) and white (i.e. Spectro < 0.28; *N* = 10) in all Swiss individuals (regardless of their *MC1R* genotype) and (ii) rufous (i.e. Spectro > 0.28; *N* = 8) and white (i.e. Spectro < 0.28; *N* = 10) in MC1R_VV_ Swiss individuals.

### (e) Variance partition among the colour QTLs

To estimate the part of variation in plumage coloration associated with the different loci as well as the remaining unexplained additive genetic variance (*Va*), we fitted an animal model using the *R* package *MCMCglmm* (version 2.34, *R* version 4.1.1) [68]. In this model, we included as fixed predictors the genotype of individuals at the three different loci as dosage and sex. The same matrix of relatedness as the one used in the GWAS was fed to the models to estimate *Va.* Models ran for 103 000 iterations, with a burn-in of 3000 and a thinning interval of 10 (effective sampling was ≥ 9384 for all model terms). We calculated the proportion of variance (and the associated 95% credible intervals) as the mean of the posterior distribution of each term (including the residuals, *Vr*) relative to the sum of the posterior distribution of all terms (i.e. the total phenotypic variance).

### (f) Synteny between assemblies

To measure the potential linkage between the markers associated with colour in the previous sections, we looked at the position of the genes surrounding the variants in different bird genomes assembled at chromosome level, namely the chicken (*Gallus gallus,* GCA_000002315.5, [69]), the collared flycatcher (*Ficedula albicollis,* GCA_000247815.2, [70]) and the golden eagle (*Aquila chrysaetos chrysaetos*, GCA_900496995.4, [71]). We identified orthologue genes in the different assembly using the annotation of each genome available on NCBI, respectively: NCBI Gallus gallus Annotation Release 104 for *Gallus gallus*, NCBI Ficedula albicollis Annotation Release 101 for *Ficedula albicollis* and NCBI Aquila chrysaetos chrysaetos Annotation Release 101 for *Aquila chrysaetos chrysaetos*. Results of the location of these genes on the different assemblies are presented in electronic supplementary material, table S4.

## Data Availability

The raw Illumina reads for the whole-genome sequenced individuals are available in BioProject PRJNA700797, BioProject PRJNA727977 and BioProject PRJNA925445. Supplementary material is available online [72].
